# Culture and personal influences on cardiopulmonary resuscitation- results of international survey

**DOI:** 10.1186/s12910-019-0439-x

**Published:** 2019-12-26

**Authors:** Janet Ozer, Gadi Alon, Dmitry Leykin, Joseph Varon, Limor Aharonson-Daniel, Sharon Einav

**Affiliations:** 1 Department of Emergency Medicine, Faculty of Health Sciences, Ben-Gurion University of the Negev, P.O. Box 653, 84105 Beer-Sheva, Israel; 2 Shalvata Mental Health Center, Hod Hasharon, Israel and Sakler School of Medicine, Tel Aviv University, Tel Aviv, Israel; 3 PREPARED Center for Emergency Response Research, Ben-Gurion University of the Negev, Beer-Sheva, Israel; 4 Foundation Surgical Hospital of Houston, Houston, TX USA; 5 School of Public Health, Faculty of Health Sciences, Ben-Gurion University of the Negev, Beer-Sheva, Israel; 6 Intensive Care Unit, Shaare Zedek Medical Center, Jerusalem, Israel and Faculty of Medicine, Hebrew University of Jerusalem, Jerusalem, Israel

**Keywords:** Resuscitation, Ethics, Personal preferences, Religiosity

## Abstract

**Background:**

The ethical principle of justice demands that resources be distributed equally and based on evidence. Guidelines regarding forgoing of CPR are unavailable and there is large variance in the reported rates of attempted CPR in in-hospital cardiac arrest. The main objective of this work was to study whether local culture and physician preferences may affect spur-of-the-moment decisions in unexpected in-hospital cardiac arrest.

**Methods:**

Cross sectional questionnaire survey conducted among a convenience sample of physicians that likely comprise code team members in their country (Indonesia, Israel and Mexico). The questionnaire included details regarding respondent demographics and training, personal value judgments and preferences as well as professional experience regarding CPR and forgoing of resuscitation.

**Results:**

Of the 675 questionnaires distributed, 617 (91.4%) were completed and returned. Country of practice and level of knowledge about resuscitation were strongly associated with avoiding CPR performance. Mexican physicians were almost twicemore likely to forgo CPR than their Israeli and Indonesian/Malaysian counterparts [OR1.84 (95% CI 1.03, 3.26), *p* = 0.038]. Mexican responders also placed greater emphasison personal and patient quality of life (*p* <  0.001). In multivariate analysis, degree of religiosity was most strongly associated with willingness to forgo CPR; orthodox respondents were more than twice more likely to report having forgone CPR for apatient they do not know than secular and observant respondents, regardless of the country of practice [OR 2.12 (95%CI 1.30, 3.46), *p* = 0.003].

**Conclusions:**

In unexpected in-hospital cardiac arrest the decision to perform or withhold CPR may be affected by physician knowledge and local culture as well as personal preferences. Physician CPR training should include information regarding predictors of patient outcome at as well as emphasis on differentiating between patient and personal preferences in an emergency.

## Background

The reported incidence of unexpected in- hospital cardiac arrest (IHCA) ranges from 0.16 to 14% [1–3]. In such cases the physician called to attend the patient may be required to undertake instant life-support decisions with little to no information regarding the patient other than their current critical state [4–6]. The literature regarding withholding and withdrawal of life support in intensive care and internal medicine suggests that local culture significantly affects end-of life decisions, including cardiopulmonary resuscitation (CPR) [7–9]. Whether local culture is also related to spur-of-the-moment decisions in unexpected IHCA remains unknown.

The rate of attempted resuscitation following non-traumatic IHCA is highly variable, ranging between 5% in some hospitals to 65% in others [10, 11]. Most clinicians would agree that CPR is not always justifiable [5]. However, substantial variability has been shown in physician preference regarding forgoing of life support even when the physician is acquainted with the baseline condition of the patient and the terminal event is expected [6, 12]. There are almost no international comparisons of physician willingness to forgo CPR in IHCA when faced with a patient they are unacquainted with. An index paper published by Richter and co-workers demonstrated that Swedish physicians chose to perform CPR in fewer cases than German or Russian physicians when presented with case scenarios of cardiac arrest [13].

Cultural differences, particularly regarding decisions that may be perceived as ethical rather than medical, may lead to conflict between medical staff members and between medical staff and their patients and/or families. Given the growth of worldwide migration, it is important to study whether such differences do exist with regards to instant decisions to withhold CPR. The current study set out to compare physicians’ attitudes towards withholding CPR for IHCA in a patient they do not know in three geographically separated and culturally-diverse countries. It was also designed to identify physician characteristics associated with the decision to forego CPR in IHCA.

## Methods

A survey was conducted among physicians who are likely to be code team members and therefore key decision-makers during unexpected IHCA.

### Study population

Physicians practicing internal medicine, anaesthesiology, cardiology, emergency medicine and critical care were surveyed in Indonesia, Israel and Mexico. These medical specialties were selected as the physicians from these departments are part of the resuscitation code team and are therefore likely to be key decision-makers during unexpected IHCA. The countries were selected due to their relative geographic, political and religious isolation from each other. This, it was presumed, would increase the likelihood of significant between-group variability in attitudes towards end-of-life issues that may be associated with the decision to withhold CPR.

### Survey method

The participants in the current survey constitute a convenience sample of physicians approached at locations such as staff meetings in departments of internal medicine, anesthesiology and cardiology (meeting topics unrelated to resuscitation) or international Intensive Care or Anaesthesiology conferences. Questionnaires were distributed among the attendees by medical students on location and were offered in English, Hebrew, Spanish and Arabic. Consistency of the non-English versions of the questionnaires with the original English version was validated through translation and back-translation. All questionnaires were completed and returned to the students on location. At least one of the co-authors attended the conference or the meeting in order to monitor and supervise the data collection as well as the integrity of the study course.

### Questionnaire structure

The process of construction and testing of the questionnaire has been described elsewhere [14]*.* In brief, the questionnaire included the respondents’ demographic details and professional expertise and experience, their approach towards patient autonomy, justice, beneficence and non-maleficence in the context of withholding and withdrawal of cardiopulmonary resuscitation, their ethical preferences (e.g. personal and patient-related attitudes towards sanctity vs. quality of life) and a series of questions designed to assess the depth of their knowledge of cardiopulmonary resuscitation. The questions constructed for the purpose of this study (i.e. those that did not belong to previously validated questionnaires) underwent three stages of development in order to achieve a final fair-to-good reliability coefficient. In order to verify standardization, a page detailing the definitions of the terms used throughout the questionnaire.

### Ethics and consent to participate

Informed consent was implied by questionnaire completion in accordance with local Institutional Review Board requirements (Hadassah Medical Centre’s Institutional Review Board which was the workplace of the PI at the time, Jerusalem, Israel). The study goals were explained both at the time of questionnaire distribution (face-to-face, verbally, based on a pre-rehearsed script) and during questionnaire completion (in writing, on the first page of the questionnaire). It was made clear to participants, in the same manner, that completing the survey questionnaire would be interpreted as agreement to participate in the study. The written introduction to the questionnaire also included documentation of the researchers’ commitment to maintain respondent confidentiality.

### Outcome measures

The primary outcome measure was the physicians’ attitude towards forgoing any attempt to perform resuscitation. The response to the following question “Have you ever decided solely based upon your own judgment, without consulting any other medical professional (doctor, nurse, or paramedic), to **not begin** resuscitation on a patient that you didn’t know? “ was used as reference for comparison. Secondary outcomes included the association between the responses and respondent characteristics.

### Statistical analysis

After describing the characteristics of the participants in the three countries, the Chi-square test was used to perform frequency analyses on categorical variables to determine whether the observed frequencies were significantly different from the expected frequencies. The non-parametric Kruskal-Wallis one-way analysis of variance was used to examine continuous variables since the assumption of normality was violated after conducting the Kolmogorov-Smirnov test. Pairwise Mann–Whitney U-tests were used to explore differences where the Kruskall-Wallis test had yielded a significant finding. Differences between related measures (e.g. assessment preference for sanctity vs. quality of life for the patient or self) were examined using the Wilcoxon signed-rank test. Multivariate logistic regression was used to examine the variables that characterize the physician who would forgo CPR (“enter” approach). Variables mentioned in the research questions were included as predictors (e.g. country) as well as variables that were found significant (*p* <  0.05) in the preliminary tests (Chi-square, Kruskal-Wallis). Reference values were determined arbitrarily. Significant effects for categorical variables were later subjected to post-hoc analysis using sequential Bonferroni correction. The results of the logistic regression analysis were tabulated and presented with the Odds Ratios, their 95% confidence intervals and *p* values for the variables tested.

## Results

Of the 675 questionnaires distributed, 617 were completed and returned (overall response rate 91.4%). Respondents were mostly secular males with children (Table 1), who had spent an average of 14.8 ± 10.5 years practicing medicine and were usually trained in anaesthesiology, intensive care or emergency medicine (61%).

**Table 1:** Demographic, professional and personal characteristics of the study population, as a whole and per country

Characteristic	Category	Israel *n* (%^a^)	Indonesia *n* (%^a^)	Mexico *n* (%^a^)	Total *n* (%^a^)	*p* value
Gender	Male	131 (65.5%)	186 (63.9%)	67 (59.8%)	384 (63.7%)	0.602
Religion	Jewish	162 (83.1%)	0	0	162 (26.9%)	N/A
	Muslim	16 (8.2%)	173 (58.8%)	0	189 (31.4%)	
	Christian	8 (4.1%)	95 (32.3%)	106 (93.8%)	209 (34.7%)	
	Other	9 (4.6%)	26 (8.8%)	7 (6.2%)	42 (7.0%)	
Degree of religiosity	Secular	132 (65.3%)	176 (60.3%)	60 (53.6%)	368 (60.7%)	0.222
	Observant	35 (17.3%)	48 (16.4%)	25 (22.3%)	108 (17.8%)	
	Religious/orthodox	35 (17.3%)	68 (23.3%)	27 (24.1%)	130 (21.5%)	
Parental status	Not a parent	38 (18.9%)	42 (14.4%)	24 (21.4)	104 (17.2%)	0.185
	Has children	163 (81.1%)	249 (85.6%)	88 (78.6%)	500 (82.8%)	
Medical specialty	Anesthesiology, Intensive Care and Emergency medicine	110 (54.7%)	178 (61.2%)	79 (71.8%)	367 (61.0%)	0.006
	Other^b^	91 (45.3%)	113 (38.8%)	31 (28.2%)	235 (39.0%)	
Time of last ACLS course	Never or more than 2 years	140 (69.0%)	201 (68.0%)	53 (47.3%)	394 (64.9%)	< 0.001
	Less than 2 years	63 (31.0%)	91 (31.2%)	59 (52.7%)	213 (35.1%)	
Prefer to avoid risks	Disagree	52 (26.1%)	79 (27.1%)	45 (40.2%)	176 (29.2%)	0.042
	Agree or don’t know	147 (73.9%)	213 (72.9%)	67 (59.8%)	427 (70.8%)	
Continuous variables	Israel Med (IQR, min-max)	Indonesia Med (IQR, min-max)	Mexico Med (IQR, min-max)	Total Med (IQR, min-max)	*p* value	
Age (years)	40 (16, 26–69)	39 (17, 26–78)	37 (16, 25–78)	39 (17, 25–78)	0.955	
Years practicing medicine	13 (18, 1–44)	11 (17, 1–44)	11 (17, 2–44)	12 (18, 1–44)	0.619	
Years practicing emergency medicine	3 (11, 0–32)	3 (9, 0–32)	2 (3, 0–32)	3 (9, 0–32)	0.092	
Knowledge score (Scale 1–5)	2 (1, 1–5)	2 (1, 1–5)	3 (1, 1–4)	2 (1, 1–5)	0.979	
Patient oriented assessment of the preference for sanctity vs. quality of life^1^ (Scale 1–5)	3 (2, 1–5)	3 (2, 1–5)	3 (3, 1–5)	3 (2,1–5)	0.005	
Self-assessment of the preference for sanctity vs. quality of life^1^ (Scale 1–5)	3 (1, 1–5)	3 (2, 1–5)	5 (1, 2–5)	4 (1, 1–5)	< 0.001	

^a^Percentages represent the proportion within each country. Different categories may thus have different percentage due to missing data

^b^ Internal Medicine, General Surgery, Pediatrics

*Note:* ^1^higher rating on this scale indicates preference for quality of life

Decimal values greater than or equal to 0.5 were rounded to the closest whole higher value. Decimal values less than 0.5 were rounded to the closest whole lower value

### Comparison of the background characteristics of the three study groups (Indonesia, Israel and Mexico)

The response rates ranged from 98.3% in Indonesia (295/300) through 86.9% in Israel (213/245) to 83.8% in Mexico (109/130)*.* The participant groups differed significantly in several background characteristics, including religious affiliation, attitude towards risk-taking behavior, professional expertise and time of last Advanced Cardiovascular Life Support (ACLS) training (Table 1). Mexican respondents placed greater emphasis on quality of life compared to both Israelis and Indonesians for themselves and for their patient (*p* <  0.01 for both, higher rating on this scale indicates preference for quality of life) (Table 1).

### Responses to the question regarding non- initiation of CPR

Among the 617 study participants, 158 (25.6%) responded that they had in the past decided to not begin CPR in a patient they do not know without ever consulting with someone who is familiar with the patient. In general, physicians who were more religious/orthodox and were less risk-averse had higher tendency to forgo initiation of CPR.

The proportion of physicians who stated that they had decided to forgo initiation of CPR was significantly higher in Mexico (34.9%) than in Israel (22.1%), *p* <  0.040. Indonesia did not differ significantly from either Israel or Mexico.

Among male participants, Mexicans were more likely (40.3%) to forgo initiation of CPR compared to Indonesian (25.8%) and Israeli (22.9%) participants. In Mexico, physicians who were not parents tended to forgo CPR more than those who had children and more than their counterparts from other countries (Table 2). Mexican participants who had completed their ACLS training more than two years prior to participating in the survey or had never completed such ACLS training also tended to forgo CPR more than those who had completed their training more recently and more than their counterparts in other countries. No country differences were noted for women, parents, and participants who completed their ACLS training two years prior to participating in the survey.

**Table 2:** Characteristics of physicians who responded that they had in the past decided to forgo initiation of CPR

Characteristic	Category option	Israel	Indonesia	Mexico	Total	
		*n* (%)	*n* (%)	*n* (%)	*n*	*p* value
Gender	Female	13^a^ (19.1%)	24^a^ (23.1%)	11^a^ (26.8%)	48 (22.5%)	0.197
	Male	30 ^a^ (22.9%)	48 ^a^ (25.8%)	27 ^b^ (40.3%)	105 (27.3%)	
Degree of religiosity	Secular	25^a^ (19.1%)	40^a,b^ (22.9%)	20^b^ (35.7%)	85^a^ (23.5%)	0.035
	Observant	6^a^ (17.1%)	13^a^ (27.1%)	6^a^ (24.0%)	25^a^ (23.1%)	
	Religious/orthodox	13^a^ (37.1%)	20^a^ (29.4%)	12^a^ (44.4%)	45^b^ (34.6%)	
Parental status	Not a parent	4^a^ (10.5%)	9^a^ (21.4%)	13^b^ (54.2%)	26 (25.0%)	0.847
	Has children	39^a^ (24.9%)	64^a^ (25.8%)	25^a^ (29.8%)	128 (25.9%)	
Medical specialty	Anesthesiology, Intensive Care and Emergency medicine	23^a^ (27.4%)	36^a,b^ (22.9%)	23^b^ (37.7%)	82 (27.2%)	0.301
	Other^*^	20^a^ (17.2%)	35^a^ (26.3%)	14^a^ (31.1%)	69 (23.5%)	
Time of last ACLS course	Never or more than 2 years	32^a^ (23.0%)	55^a^ (27.5%)	23^b^ (44.2%)	110 (28.1%)	0.079
	Less than 2 years	12^a^ (19.0%)	18^a^ (19.8%)	15^a^ (26.8%)	45 (21.4%)	
Prefer not to take risks	Disagree	14 ^a^ (26.9%)	24^a^ (30.4%)	19^a^ (42.2%)	57 (32.4%)	0.014
	Agree or don’t know	29^a^ (19.9%)	47^a^ (22.2%)	19^a^ (30.2%)	95 (22.6%)	
Characteristic	Israel Med (IQR, min-max)	Indonesia Med (IQR, min-max)	Mexico Med (IQR, min-max)	Total Med (IQR, min-max)	*P* value	
Age	40 (17, 28–66)	39 (15, 28–69)	36 (19, 28–69)	39 (15, 28–69)	0.955	
Years practicing medicine	16 (17, 1–40)	12 (18, 1–44)	11 (23, 3–44)	12 (17, 1–44)	0.619	
Years practicing emergency medicine	8 (18, 0–30)	5 (8, 0–30)	2 (5, 0–30)	4 (10, 0–30)	0.092	
Knowledge score (Scale 1–5)	3 (1, 1–5)	2 (1, 1–5)	3 (1, 1–4)	3 (1, 1–5)	0.979	
Patient oriented assessment of the preference for sanctity vs. quality of life^1^ (Scale 1–5)	3 (2, 1–5)	3 (2, 1–5)	3 (2, 1–5)	3 (2, 1–5)	0.005	
Self-assessment of the preference for sanctity vs. quality of life^1^ (Scale 1–5)	3 (2, 1–5)	3 (2, 1–5)	5 (0, 3–5)	4 (2, 1–5)	< 0.001	

*Note:* While 158 participants indicated they would not initiate CPR, total number of participants on different background characteristics may vary due to missing data on these variables

Numbers within parenthesis indicate percentages of participants deciding not to initiate CPR within each background characteristic. ^a,b^ indicates statistically significant differences (*p* < .05) between countries in characteristic’s category, using the Z-test for independent groups proportions. *P* indicates Chi-Square or Fisher (2X2 tables) of characteristic’s category comparison regardless participant country

^*^ Internal Medicine, General Surgery, Pediatrics

*Note:* ^1^higher rating on this scale indicates preference for quality of life

Decimal values greater than or equal to 0.5 were rounded to the closest whole higher value. Decimal values less than 0.5 were rounded to the closest whole lower value

Among secular participants, Mexicans were more likely to forgo initiation of CPR (35.7%) compared to Israeli participants (19.1%), while Indonesian participants did not differ from either. No significant differences between countries were observed for observant and religious/orthodox participants. However, within Israel, affiliation of the participants to the religious/orthodox group was associated with a positive response to the reference question.

Regarding medical specialty, Mexican participants from anesthesiology, intensive care and emergency medicine were significantly more likely to forgo initiation of CPR (37.7%) compared to Indonesian participants (22.9%) but not to Israeli participants in the same fields of expertise.

Country differences in continuous background variables among participants who decided to forgo initiation of CPR in a patient they do not know are presented in Table 2. Mexican responders placed higher emphasis on quality of life when requested to grade self-preferences regarding quality of life vs. sanctity of life, as opposed to their colleagues in Israel and Indonesia (*p* <  0.001, higher rating on this scale indicates preference for quality of life) (Table 2).

### Multivariable logistic regression modeling of the characteristics associated with physicians declaring that they had decided to forgo initiation of CPR efforts

Physician age, medical training and experience (i.e. specialty and resuscitation training) were not found to be associated with the decision to forgo CPR. Conversely, country of practice, level of knowledge about resuscitation and degree of religiosity were strongly associated with the decision to forgo CPR. Mexican physicians were almost twice more likely to forgo CPR than their Israeli and Indonesian counterparts [OR 1.84 (95% CI 1.03, 3.26), *p* = 0.038]. Increasingly greater theoretical knowledge of resuscitation was associated with an increasingly higher probability of being willing to forgo CPR [per each additional knowledge point OR 1.29 (95% CI 1.04, 1.61) *p* = 0.022]. The variable most strongly associated with avoiding CPR was the degree of the participants’ religiosity; orthodox respondents were more than twice more likely to be willing to forgo CPR than secular and observant respondents, regardless of the country of practice [OR 2.12 (95%CI 1.30, 3.46), *p* = 0.003] (Table 3).

**Table 3:** The characteristics associated with a physician declaring they ever decided to forgo initiation of CPR

Variable	OR (95% CI)	*p* value
Country:		
Israel vs. rest	0.54^a^ (0.31, 0.97)	0.038
Indonesia vs. rest	0.57^a^ (0.34, 0.95)	0.033
Mexico vs. rest	1.84^b^ (1.03, 3.26)	0.038
Age	0.99 (0.95, 1.03)	0.555
Knowledge Test	1.29 (1.04, 1.61)	0.022
Medical Specialty	1.01 (0.67, 1.51)	0.981
Time of last ACLS course^1^	0.66 (0.42, 1.06)	0.083
Degree of religiosity:		
Secular vs. rest	0.47^a^ (0.29, 0.77)	0.003
Observant vs. rest	0.39^a^ (0.2, 0.76)	0.005
Religious/orthodox vs. rest	2.12^b^ (1.30, 3.46)	0.003
Years practicing medicine	1.03 (0.99, 1.08)	0.132
Preference not to take risks^2^	0.69 (0.45, 1.08)	0.088

Note: ^1^Time of last ACLS course was compared as follows: never or more than 2 years vs. less than 2 years. ^2^ Personal preferences to risk taking was compared as follows: disagree vs. don’t know & agree

^a,b^ indicate a pairwise comparison using the Bonferroni correction. The χ^2^ = 32.52, *p* < .001, Cox & Snell R Square = .059, − 2 Log likelihood = 593.92

## Discussion

The present study shows that in unexpected IHCA, when the physician is not acquainted with the patient, decisions regarding CPR may be driven by local culture and probably by some of the characteristics of the physician on location.

The current Criteria for Not Starting CPR (AHA guidelines 2010, part 3 “Ethical Issues”) [15] include patients with valid Do Not Attempt Resuscitation (DNAR) order and indisputably irreversible death. By extrapolation, all other patients who suffer cardiac arrest in the hospital setting should undergo CPR. This criteria were not reviewed in 2015 AHA guidelines, and the current updates refers only to limitation of interventions and withdrawal of life-sustaining therapies in adult patients [16].

From a study that sought to identify the effectiveness of interventions to increase Advance Directive completion rates in the US, it arises that despite federal and state laws governing Advance Directives, the pre intervention completion rate of Advance Directives was below 20% [17]. Another study conducted in Belgium presents that of all the respondents, only 4.4% had spoken to their physician about their wishes regarding end of life medical treatment [18]. Given the fact that in the countries included in the study there is no official policy of intervention in order to increase the awareness to Advance Directives, the majority of the physicians rely on their own judgment.

Best available evidence should guide clinical decisions regarding the care of individual patients. Our findings suggest that, in the absence of evidence based recommendations, a subjective decision driven by variables other than the best interest of the patient may drive CPR decisions for many cases of IHCA.

In this study, the geographically and culturally-distinct physician populations provided a unique platform for comparing responses to issues that may have been affected by culture, but may also be related only to the normal distribution of any human population with modern medical training. Additional advantages in this study include the selection of a respondent population that comprises the major medical disciplines involved in resuscitation and the fact that physicians from multiple medical centres were surveyed which lends greater generalizability to the findings.

Our findings imply that personal preferences for quality of life over sanctity of life may be associated with the decision to perform CPR when no background information is available regarding the patient. Physicians from Mexico placed quality of life over sanctity in majority of the statements regarding themselves and were almost twice more likely to forgo CPR than their Israeli and Indonesian colleagues. This finding correlates with studies showing that physicians’ perceptions of their patients’ wishes for treatment are influenced by what they would want for themselves [19].

Decisions to forgo CPR may also be influenced by institutional policy [20] and personal beliefs and education. In the current study decisions regarding performance of CPR varied significantly between culturally and geographically distinct countries. Although physicians with a tendency towards avoiding CPR did seem to have some common characteristics regardless of country (e.g. more years of experience in medical practice in general and in emergency medicine in particular, and older age), in the univariate analysis, these findings did not reach statistical significance in the multivariable logistic regression model. The degree of religiosity was found to be most significant factor in the examined data set, remaining significant even after adjustment for other variables.

End-of-life decisions of elderly Jewish populations often adhere to the ancient Talmudic concept which argues that when death is inevitable it should not be interfered [21]. Reluctance to interfere with the dying process may override western medical culture where, lacking a clear do-not resuscitate order, physicians and nurses are generally obligated to provide CPR [21]. However, these finding contrasts with previous studies that showed that physicians who believe that death is determined by a higher power tend to provide more aggressive treatment at the end of life situations [22, 23].

The finding that Israeli medical professionals (that in current study were predominantly Jewish and secular) were less willing to forgo CPR than their Mexican colleagues is in line with previous reports. Bülow and colleagues [24] examined attitudes towards treatment of terminally ill or permanently unconscious patients. Protestant and Catholic professionals were more willing to withhold a potentially lifesaving treatment in accordance with (previously competent) patient request than their Jewish counterparts (84, 73 and 67% respectively). Another study, conducted in Israel, revealed high rates of out-of-hospital resuscitation (67.5%) despite common presentation of asystole as first presenting rhythm (76.3%) [25] .

In a discussion of medical explicit and tacit knowledge in complex circumstances Brummell et al. [26], note that in emergency situations, where there is no time to take a medical history, decisions are often made based on obvious signs such as perceived patient age and presenting cardiac rhythm and on personal experience of CPR success rates. In the current study, tacit medical knowledge played a significant role in physician willingness to initiate CPR efforts whereas years practicing medicine was not. This is unsurprising given that most physicians are more likely exposed the unrealistic expectations of CPR outcome created by multiple media sources [27–30] than to professional literature on the topic.

Performing CPR may be much easier than avoiding action, if resuscitation is perceived as a transition phase, softening the event of sudden death. Especially when the physicians experience discomfort while facing end of life decisions [31]. Additional reasons for implementing CPR indiscriminately include insufficient professional knowledge, limited physician-patient communication, guilt and treatment requests by patients or families [32, 33]. It has been shown that family members of terminally ill cancer patients, that act like surrogate decision makers, perceive the decision as “choice between life and death”. The moral and ethical burden that forgoing CPR will be perceived by the surrounding environment, that the patient is not worth saving, led them to request to begin the resuscitation efforts [34]. In another paper, medical students, resident physicians and attending physicians were asked to estimate the rate of in-hospital cardiac arrest that survival to discharge. Nearly 50% of all residents and students and about 60% attending physicians provided inaccurate estimations [35].

All the articles mentioned above shows that personal beliefs and knowledge of medical personnel are not compatible with real practice.

### Limitations of the study

The study was based on a convenience sample and relied on self-reported data, which can be inaccurate, especially in cases of non-canonical decisions.

Some variables that could be associated with the decision to forgo CPR may have been overlooked. Not in the least, among the variables not studied were patient characteristics such as older age, mechanical ventilation, first presenting rhythm etc. that are obvious without prior acquaintance with the patient.

## Conclusions

The current study suggests that non-clinical influences may constitute an important yet largely unrecognized obstacle to the practice of evidence-based medicine in emergency decision-making regarding CPR. These findings require validation in larger and more international cohorts. Nevertheless, physician CPR training should include information regarding prognostic variables and outcomes as well as an emphasis on differentiating between patient and personal preferences in emergency situations. Further research can contribute to the better understanding of the large variance in the reported rates of attempted resuscitation following non-traumatic IHCA.

## Data Availability

The datasets generated and analysed during the current study are not publicly available due to unpublished data but are available from the corresponding author on reasonable request.

## Abbreviations

ACLS: Advanced Cardiovascular Life Support
AHA: American Heart Association
CPR: Cardiopulmonary Resuscitation
DNAR: Do Not Attempt Resuscitation
IHCA: In-Hospital Cardiac Arrest
